# Australian Catholics’ Lived Experiences of COVID-19 Church Closures

**DOI:** 10.1007/s10943-023-01823-6

**Published:** 2023-04-27

**Authors:** Philippa Martyr

**Affiliations:** School of Biomedical Sciences, University of Western Australia, M Block, QEII Medical Centre, Nedlands, WA 6009 Australia

**Keywords:** COVID-19, Catholic, Australia, Qualitative

## Abstract

In 2020, Australian Catholic churches closed due to COVID-19 restrictions, but there has been little qualitative data published on the lived experience of churchgoing Catholics in this period. Data from 175 Australian churchgoing Catholic survey participants who left responses describing their COVID-19 worship experiences as part of a larger project revealed five major themes: ‘Church and State’, ‘Blessings in disguise’, ‘Polarisation’, ‘Future proofing’, and ‘Loss’. Respondents expressed a diverse range of views about the church leadership, lockdowns, conspiracy theories, the merits and deficits of online worship, and their own thriving or suffering during church closures.

## Introduction

From 23 March to early July 2020, all churches and other places of worship in Australia were closed due to concerns about COVID-19 transmission. This included Catholic churches, where virtual worship has historically not been seen as an effective substitute for face-to-face ritual contact (Sarah, 2020). All 28 Australian Catholic dioceses and its five Eastern Rite eparchies and two ordinariates complied with this national government policy. However, because Australia’s health system is primarily a question of state rather than federal jurisdiction, COVID-19 church re-openings began unevenly across the country from early July 2020, with the state of Victoria maintaining the longest period of closure well into 2021. State-mandated church closures attracted mixed responses in the Australian Catholic media, ranging from those urging compliance and making the best of the situation (Ng, 2020; Rodrigues, 2020) to those actively opposed to what they saw as unjust impositions (Comensoli, 2020; Goulding, 2020).

During COVID-19 church closures, most Australian Catholic dioceses did not restrict the individual reception of the sacraments outside of Mass with the use of hand hygiene, distancing, and appropriate personal protective equipment (PPE). However, three dioceses (Wollongong, Canberra–Goulburn, and Wagga Wagga) imposed restrictions on individual Holy Communions, and three dioceses (Brisbane, Cairns, and Wollongong) imposed restrictions on the hearing of individual confessions (Martyr, 2022b). As churches reopened, individual dioceses introduced rules around limited numbers, social distancing, and mask-wearing in Catholic churches, according to their current state law requirements.

There are around 600,000 churchgoing Catholics in Australia—that is, those who attend Sunday Mass regularly at the rate of at least several times a month (National Centre for Pastoral Research [NCPR], 2020). This group of Catholics shows a high level of regular time investment and are the group who church closures would have impacted the most during COVID-19. They are also a shrinking group: Sunday Mass attendance rates in Australia have fallen from 15% in 2001, when national counts were introduced, to 12% in 2016 (NCPR, 2020). Clements and Bullivant (2022) have recently hypothesised that some worship behaviours of shrinking Catholic churchgoing populations may represent a ‘minority effect’ in which members of the group adopt a more consciously countercultural expression of religious beliefs.

There have been several quantitative survey-based studies of Catholic churchgoers during COVID-19 (Skalski et al., 2022; CatholicVoices, 2020; Martyr, 2022a, 2022b; Village et al., 2021). The National Church Life Survey in Australia in 2021 also captured a possible COVID-19 church closure effect when participants across several different Christian denominations were asked ‘To what extent has your faith grown in the past year through your local church?’. In 2016, 33% of participants reported a growth in faith, but in 2021 this had fallen to 25% (National Church Life Survey, n.d.).

Given the small size of the Australian churchgoing population, it is understandable that while international qualitative studies of the impact of COVID-19 on all aspects of Christian church life and worship proliferate (De Souza et al., 2021; Francis & Village, 2022; Kim, 2022; Magezi, 2022; McKenna, 2021), there are still comparatively few qualitative COVID-19 impact studies of this group of Australian Catholics. Kenkmann and Burkard (2022) qualitatively surveyed 12 Catholic older adults in Germany to assess the impact of lockdown and alternative forms of worship. They found three themes: ‘Value of church services’, ‘Alternative forms of service’, and ‘Informal spiritual and religious experiences’. Participants reported both positive and negative experiences of virtual worship, including ease of access and better quality of liturgy online, but also feeling limited by their lack of internet skills or access.

McCarthy (2021) has also presented qualitative data drawn from Australian Catholics which provides an outline of their worship experiences during church closures. She provides no total number of participants but lists several different sources: a Catholic women’s group in Perth, Western Australia; members of the Australian Academy of Liturgy; the parishioners of a Catholic parish in Brisbane, Queensland; Fr David Barry from Holy Trinity Abbey, New Norcia, Western Australia; and members of McCarthy’s own family. These participants also reported both positive and negative experiences of using different alternative forms of worship during church closures, including disappointment at opportunities lost.

This paper proposes to expand this limited literature by examining qualitative data on the lived experiences of a broad cross section of Australian churchgoing Catholics during the COVID-19 church closures in 2020. These data were collected as part of a larger study on Australian Catholic worship choices made by churchgoing Catholics during church closures. The data were captured between 22 August and 5 October 2020, when some Catholic churches were beginning to re-open in Australia, but social distancing and masks were still required in most areas, and some churches were still closed (Martyr, 2022a, 2022b).

The original study recruited a convenience sample of 1173 adult (18 +) churchgoing Catholics (39.3% males, 59.8% females, 0.9% other) nationally via Australian Catholic social media and parish networks. Participation was voluntary and anonymous, and the Monash University Human Research Ethics Committee approved the study (project number 25504). The study collected demographic and worship choice information and also administered the New Indices of Religious Orientation (NIRO) and Spiritual Wellbeing Scale (SWBS) as measures of religious orientation and wellbeing of this sample during church closures. The survey did not distinguish between lay, clergy, or religious participants (Martyr, 2022a, 2022b).

## Method

In the original study, 288 participants out of 1173 (around 25%) left responses at the end of the survey in a free text area in response to the prompt, ‘Please let us know any further responses you have about this project in the box below’. After excluding all responses that related solely to survey design and administration, praise for the project, or requests for feedback, a group of 175 respondents remained who addressed a broad range of COVID-19 and Church-related topics. Responses were downloaded from SPSS into Excel and coded manually by the author with up to five themes for each comment. The data were also uploaded to NVivo and auto coded to verify that the manual coding was accurate, detailed, and sufficiently nuanced (Welsh, 2002).

## Results

These responses, coded into five major themes, provide an opportunity to hear the voices of a group of 175 churchgoing Catholics across Australia and their lived experience of church closures. The voices are diverse and represent a broad range of experiences, both positive and negative. They come from men and women, traditionalist and liberal minded Catholics, and across a range of age groups from 18 years to 80 + years. As such, they represent the broadest and largest cross section of the churchgoing Australian Catholic public that has been captured in qualitative COVID-19 studies of this population (of which McCarthy, 2021, is the only other example at present).

Demographic characteristics of this group broadly matched the larger sample and data on Australian churchgoing Catholics for age, gender balance, national distribution across states and dioceses, Mass attendance, and country of birth (Table 1).

**Table 1:** Participant demographic characteristics

	Qualitative sample (*n* = 175)	Total sample (*N* = 1173)	2016 Australian churchgoing Catholics*
*Sex*			
Male	72 (40%)	461 (39%)	38%
Female	109 (60%)	701 (60%)	62%
Median age group (18–80 + years)	50–54 years	45–49 years	63 years
*Location by postcode*			
NSW/ACT	59 (33%)	389 (33%)	40%
Victoria	62 (34%)	367 (31%)	29%
Queensland	30 (17%)	144 (12%)	13%
Western Australia	17 (9%)	180 (15%)	11%
Other states and territories	13 (7%)	93 (8%)	7%
*Country of birth*			
Australia	139 (77%)	882 (75%)	57%
Outside Australia	42 (23%)	291 (25%)	43%
*Pre-closure Mass attendance rate*			
Less frequent	14 (8%)	64 (6%)	–
Weekly	42 (23%)	302 (26%)	–
Very frequent	125 (69%)	807 (68%)	–

^*^NCPR, (2020)

Coding revealed five major themes, in order of frequency of responses: Church and State, Blessings in disguise, Polarisation, Future proofing, and Loss. Respondents are identified by gender, age group, location in Australia by state or territory, and frequency of Mass attendance before COVID-19 church closures: ‘very frequent’ is weekly or more often, ‘weekly’ is Sunday Mass attendance, and ‘less frequent’ are those who attended Mass several times a month. I have endeavoured to present as many individual responses as possible, and to use responses which show the widest breadth of views in each theme and sub-theme. Participants’ idiosyncrasies of spelling and grammar have been preserved.

### Church and State

This was the most common theme (27 responses) and included those expressing anti-lockdown opinions and mixed attitudes towards both church and secular leadership. A small number of respondents mentioned the influence of conspiracy theories and what could be described as a perceived broader societal loss of God and religion. There were very few responses relating to the use of vaccination, masks, and social distancing.

#### Anti-lockdown Opinions

This formed the largest subgroup of responses (21 responses), which ranged from resignation to anger.

I do not believe that this virus constituted a full lockdown of religious worship, regardless of which religion you are. We should have been able to attend Masses, with all the necessary restrictions that were put in place. Depriving Catholics of their right to worship and practice their faith was wrong. Receiving Communion every Sunday is the most important part of our religion and faith. The government broke our constitutional implied right by taking that away from us. (Female, 18–24, South Australia, very frequent)

While I support and fully comply with the restrictions in Victoria, I feel that the restrictions on worship are heavy handed and a breach of the separation of church and state. I think the government should have issued clear guidelines for churches on how to be COVID safe, but should not have banned any public worship, especially Mass, or any private prayer in churches. (Male, 50–55, Victoria, very frequent)

I think that there is no excuse for the Church to ever succumb to government enforced lockdown again. (Male, 30–34, Queensland, weekly)

There was resentment of perceived inconsistencies between the regulation of church worship and other activities.

If [people] can gather for sport games or go to the shops they also should be allowed to gather for Mass, with necessary safety measure in place. (Female, 40–49, Victoria, very frequent)

I am frustrated that can have 50 people at home without supervision, but unable to go to Mass without the strict cleaning/marshalling/etc in place—prefer to stay at home and watch Mass in the lounge room. (Female, 65–69, South Australia, very frequent)

#### Mixed Attitudes Towards Church and Secular Leadership

Another large subset of responses on church and secular leadership (14 responses) ranged from resignation to anger.

I think it is a real shame that the Church hierarchy have kowtowed so much to the authorities in closing churches, stopping the sacraments and even refusing sometimes to give sacraments to the dying during this pandemic. Look at what brave Christians have done throughout the centuries to bring Christ to people – against all odds. And now look at what our churches have done during this time. (Female, 50–54, NSW, very frequent)

The bishops have let us down. Weak men, completely useless. (Male, 45–49, Queensland, very frequent)

I do wish the bishops might have shown a little more leadership during this crisis—they seem to be irrelevant and ignored by the media. (Female, 50–59, Victoria, very frequent)

However, some respondents expressed support for both church and secular leadership, and for lockdown restrictions.

Bishops advocating social distancing and hygiene measures to enable church worship to occur are to be thanked. (Male, 55–59, NSW, weekly)

I think until the pandemic is over there should be limited and tightly controlled opportunities for Mass. There are other ways to connect with God that does not put the community at risk. (Female, 55–59, NSW, very frequent)

I hope and pray that churches will open for services but in conformity with the COVID rule like distancing, mask wearing, etc. (Male, 65–69, Victoria, very frequent)

#### Conspiracy Theories

No respondents shared views that could be categorised as conspiracy theories related to the origin and transmission of COVID-19 or protective measures taken against its spread in the community. However, several respondents shared feelings of frustration at the influence of conspiracy theory thinking among friends and relatives.

Devout Catholics in Australia, at least in my circles, are going deeper and deeper into crazy conspiracy territory with fringe online internet consumption and I no longer have anything of substance to relate to anymore yet alone to talk about the day-to-day things in life. My parents no longer talk to me about anything except global conspiracy theories and apocalyptic predictions of the end times. COVID-19, if anything, brought these themes to a head as I witnessed so many Catholics I know disregard the advice of parish priests, government directions, health authorities, and just general safety precautions of the common good for the sake of piety. (Male, 25–29, NSW/ACT, weekly)

One of the most demoralising aspects of COVID has been watching Catholics promote conspiracy theories and demand their ‘right’ to worship without caring for vulnerable persons. (Male, 45–49, NSW/ACT, very frequent)

Too often, we see some individuals whose quite rational wariness and distrust of government in religious matters has spilled over into conspiracy theories. This can make it very difficult to deal with a few individuals who attend worship while rebelling against the very hygiene measures which enable attendance to occur safely. (Male, 55–59, NSW/ACT, weekly)

#### Broader Societal Loss of Faith in God

This subset of responses (7 responses) described a perception of a broader loss of religious faith and practice. The only connection made between societal loss of faith in God and the pandemic was that this changed social context made life harder for Catholics to live as Catholics.

Personally, I believe a lot of today’s societal problems stem from not having a relation with God. I believe that religion is used for aesthetic purposes and not truly and wholly for the right reasons. A lack of God and faith leads to a lack of reason and purpose. (Female, 25–29, Queensland, very frequent)

To live a good Catholic life has become much harder in the past 20 years. Due to the breakdown in moral standards. The world has become a place where anything goes. There is no longer right and wrong. God’s standards no longer matter and sadly most politicians run with this. (Male, 75–79, NSW/ACT, very frequent)

### Blessings in Disguise

This was the second largest theme (19 responses) and included responses reflecting positively on the overall COVID-19 experience. These included reflections on personal faith in God in times of adversity, examples of faith enrichment during COVID-19, and positive experiences with online worship.

#### Personal Faith in God in Adversity

Some respondents (13 responses) took the opportunity simply to praise God in their responses, while others provided more detail about how present suffering was clarifying their faith.

If I didn’t have God I would have broken down. I actually had multiple impossible situations happen on the lead up to COVID and then financial uncertainty and other difficulties made it unbearable. If I did not have God and an understanding of God’s care for me, then I would have literally had a break down. God actually gave me meaning for the suffering through personal revelation. It was only after that day that I started to recover. (Female, 45–49, NSW/ACT, very frequent)

Things happen for a reason as part of God’s mighty plan though we may not be able to comprehend sometimes why things happen. For our part we carry on trusting in Him. (Female, 55–59, Victoria, very frequent)

I felt strongly that if I not had God in my life years before that happening then life would have been more difficult than it already had been. (Female, 80+, Western Australia, very frequent)

#### Faith Enrichment During COVID-19

Seven respondents found that lockdown gave them new opportunities and perspectives on their faith and worship which they found personally enriching.

During COVID closures, I began praying the Divine Office much more frequently to give shape and structure to my daily life. (Male, 30–34, NSW/ACT, very frequent)

Because of the COVID restrictions I have a newfound appreciation for the Mass, the Eucharist and the priesthood. … I have made a commitment to going to daily Mass more often and praying for priests more often. (Female, 60–64, Western Australia, very frequent)

As an aged person living alone, but always busy with ‘good works’, I feel that the isolation period was like a religious retreat, giving me time to catch up on spiritual reading, write a daily journal, appreciate the good things about my life. (Female, 80+, Queensland, very frequent)

#### Positive Experiences with Online Worship

Respondents also reported a range of positive experiences of online worship, sometimes to their personal surprise (15 responses). Many became aware for the first time of online faith resources apart from worship which they also found helpful.

I like internet access to Mass so much I now watch a recording every day. Not having to travel to a town is a bonus. Being a strong introvert, I never feel lonely or feel the need to belong to a community. By attending Mass on the internet I have come across links to other online videos which gave my spiritual life a great lift and resulted in me buying about 6 spiritual books, and really changed my prayer life. (Female, 65–69, Victoria, weekly)

When our churches closed, I was very disappointed in our churches Mass streaming so, I took the opportunity of surfing the net and found and enjoyed many other ‘productions’. I eventually ‘joined’ another diocese live streaming and was very happy. (Female, 75–79, NSW/ACT, very frequent)

Another response is access to so much online nourishing spiritual retreats, webinars, Youtubes, podcasts through the likes of Word on Fire ministries, Ascension Press, etc that I have become aware of during this period. Fantastic resources and so available and current and timely. Very needed in our spiritually dry Australian context. (Female, 50–59, NSW/ACT, weekly)

### Polarisation

This theme encompassed responses where respondents expressed views on broader Church politics and liturgy outside of COVID-19. Of all responses where a clear liberal or traditionalist Church view was presented (20 in all), 15 came from those with liberal views, most of whom were female, and the largest age group was those aged 80 + years. Those with more traditionalist views were much younger (18–24 years) and more likely to be male.

Nine responses directly reflect a liberal/conservative divide related to COVID-19 church closures—that is, a divide between those who prefer a less hierarchical church and lobby for changes, such as the inclusion of women in the ordained ministry, and those who prefer traditional Catholic belief and teaching in all aspects of Church life (Coady, 2015; Starks, 2013). This theme includes responses about the use of the Latin Mass, a perceived loss of voice for faithful Catholics, suspicion about the survey and how its results would be used, and expressions of broader discontent with mainstream Catholicism.

#### The Latin Mass

A subset of participants in the original study expressed preferences for the traditional Latin Mass, and this was reflected in some responses.

COVID-19 restrictions have pulled me closer to God, and I am now attending the Latin Mass. (Male, 20–24, NSW/ACT, weekly)

I’m in a complicated situation with my faith, but when I attend the Latin Mass regularly, I feel a lot better. I don’t get the same senses from other forms of worship. (Female, 18–24, Victoria, very frequent)

Our Lord has used this experience to draw me and my family back to those priests and parishes who celebrate the Traditional Mass and follow the Traditional way of living and preaching the Catholic Faith. … I will never darken the doors of another Novus Ordo liturgy as long as I live. (Male, 40–44, Western Australia, very frequent)

However, some respondents were less enthusiastic:

I attended Latin Mass and there was no social distancing—queuing for communion was very closely packed. There were more attendees than mandated. Communion was taken on the tongue (I did not partake) without any cleansing between parishioners. There was no marking on the pews for social distancing and no hand sanitiser. I have not been back since, although my husband and children have. (Female, 60–64, NSW/ACT, very frequent)

#### Loss of Voice

Five responses reflect a sense of disenfranchisement, especially of traditional-minded Catholics. COVID-19 and its lockdowns are not seen as the cause of this, but rather as expressions and symptoms of a deeper problem.

I have a sense of foreboding in that I expect far more persecution of the Church worldwide—burning of churches etc and being forcibly excluded from being able to express Catholic views in the public sphere. The example of churches not being considered as essential services by politicians, which kept them closed, when other more frivolous venues were being opened is an example of how the Church is being silenced and suppressed. (Female, 60–64, Western Australia, very frequent)

… while it is fulfilling and meaningful for me to be a practicing and devout Catholic, I think it’s something that is becoming increasingly difficult as we delve further into a secular society and because of that there is a lot of despair … I feel I’m up against the world whether it be my peers, the content of my subjects or the general culture of society, it’s very hard to speak my mind. (Male, 18–24, Victoria, weekly)

I also believe people are often silenced with their religious beliefs, probably more so Roman Catholics due to past scandals in the Church, thus this has led to people being quiet about their faith or even leaving because they don’t want to be seen a ‘Catholic’. (Female, 25–29, Queensland, very frequent)

#### Suspicion

Three respondents expressed suspicion about the intentions of the original survey project and about how its data would be used. These came from both traditional Catholic and more liberal Catholic perspectives and reflect a broader Catholic culture of suspicion about a researcher or writer’s ‘political’ position in the Church (Starks, 2013).

Why are you doing this research. Are you pro church or against church or neither? (Female, 55–59, NSW/ACT, very frequent)

Having suffered enormous bigotry prejudice discrimination hatred and actual physical emotional and psychological harm and disadvantage throughout my life because of my Catholic identification from Protestants atheists and non-religious I am wondering whether this survey will be used against Catholics and to harm us—even more so from the current and increasing anti-Christian anti-Catholic environment. (Female, 75–79, Victoria, very frequent)

Perhaps there is an interesting and somewhat conservative/traditional theology that underpins this survey: God as external operator over one’s life. I don’t subscribe to this theology ... (Female, 70–74, South Australia, very frequent)

#### Broader Discontent

These responses reflect a range of views of dissatisfaction with the Church in Australia, both theologically and organisationally. Some of these responses related to the concerns of liberal-oriented Catholics and to issues that began before the pandemic.

I felt absolutely let down by my church before the pandemic, so nothing has changed now. My spiritual nourishment during the pandemic has come from spiritual reading and discussions with friends. My parish, in recent times, has done nothing to nourish my faith and, in fact, has caused me to feel very depressed about the role of the church in the world. I would like to see some move towards reform on many levels. I think the church is doomed if this does not happen. (Female, 75–79, NSW/ACT, very frequent)

To me, my ‘religion’ is separate from my ‘faith’. My faith in God and in His word (Bible scripture) is unwavering. However, my connection with the (Catholic) Church, my ‘religion’ is weakened by some actions/decisions of the Church. For example, I believe the Catholic Church’s handling of child abuse in the Church has been appalling. Also, some of the Catholic Church’s mandates, such as clerical celibacy, are archaic. I have been raised a Catholic, but more increasingly I am finding encouragement and enlightenment in other Christian ministry. (Female, 50–54, Queensland, very frequent)

My relationship with the Catholic Church was irrevocably damaged by the Church’s response to the pandemic. … As a result, I intend to join another Christian ecclesial community in the near future, having experienced the lengths to which they went to minister to members during the pandemic. (Female, 70–74, Western Australia, very frequent)

However, there were other responses that related to traditionalist concerns.

I have started questioning being Catholic with priests and nuns telling me and others gay marriage etc is fine. Not going to confession or going to Mass is fine. What there bringing into the school curriculum regarding sex. Also a nun just told me in some situations abortion is ok. Priest aren’t giving direction in the sermons. I have to go and look for strength on the Internet. (Female, 60–64, NSW/ACT, very frequent)

I have three local choices for weekend Mass. One parish has a womanish, long-winded and left-wing priest, the second seems in thrall to a deacon whose tedious and lengthy ‘sermons’ consist mainly of folksy, self-serving chitchat and a third, whose bizarre incumbent frequently preaches heresy. I am not a parish-person and probably never will be. However, I persevere in attending Mass, with the belief that God is in charge. (Female, 60–64, Western Australia, very frequent)

### Future Proofing

Some respondents (14 responses) made suggestions as to how a future pandemic could be handled in terms of access to worship. Mass was the most frequently mentioned sacrament.

#### The Mass

Missing Mass and being denied Communion was a key concern for respondents, even with the dispensation from Sunday Mass attendance in place across Australia. This drove responses about how this could be prevented in the future.

Next pandemic and NOW for Melbourne why can’t we attend Mass sitting in our cars in church car park—wear masks and gloves to receive communion brought to car. Bunnings [hardware store] has click and collect, why can’t churches. (Female, 70–74, Victoria, very frequent)

It would have been nice for archbishops and bishops alike to have allowed ‘drive-in’ Masses as seen in the US. (Male, 18–24, Victoria, very frequent)

I really missed not being at personally at Mass during the lockdowns and would prefer to take on the risk of being next of a sick person, rather than not receiving the Eucharist at all. (Female, 45–49, NSW/ACT, very frequent)

#### Confession

Second only to the Mass were responses about the inability to access the sacrament of Confession easily.

During the lockdown, I would have liked to see parishes make a greater effort to offer confessions. (Male, 25–29, NSW/ACT, very frequent)

I miss the sacrament of confession (even if spiritual confession can take its place—given the situation). (Female, 55–59, NSW/ACT, very frequent)

I wanted to be able to go to confession (Sacrament of Reconciliation) but had no way to. I understand why this is, from a public health viewpoint, but our spiritual life is what is most important at these times and I was truly feeling the absence of the sacraments when I needed them the most. (Female, 45–49, Victoria, very frequent)

#### Other Pastoral Support

Respondents offered responses for and against the level of general pastoral support and clergy outreach they had experienced.

I have found the pastoral outreach of the parish poor during Covid-19 lockdown in Vic. I have received just one phone call from the [parish priest] in the past 6 months and that was when my wife’s sister died. … It begs the question what pastoral training do priests get in their formation to deal with lockdowns like this? (Male, 75–79, Victoria, weekly)

I am aware of a few priests who have been willing to meet people in parks and walking tracks to give spiritual direction and hear confession and even to visit people at home and give Communion and the last rites. But many priest have not, including my own parish priest who has basically locked himself away from everyone for months because he is scared of coronavirus. (Male, 55–59, Victoria, very frequent)

The personal touch from parish would have made live streaming feel more connected. i.e. phone calls from the priest. (Female, 55–59, South Australia, very frequent)

### Loss

This final theme is related to the theme of ‘Future proofing’, and there was some overlap between these two themes. Respondents shared their personal experiences of hardship and exclusion during COVID-19 church closures, described how their faith and religious practice suffered, and how online worship did not meet their deeper spiritual needs.

#### Hardship and Exclusion

Respondents expressed personal difficulty and hardship at the sense of being excluded from church worship.

Not being able to attend Mass is torturing for me. Church should be considered essential during lockdown. It is bad for mental health. Receiving the sacraments is like life’s balm. (Female, 45–49, Victoria, very frequent)

I feel really sad and I still can’t believe that we can’t go to church. We need our spiritual food same as food from supermarkets. Online Mass is not enough... (Female, 35–39, Victoria, very frequent)

… I think the hardest thing about the pandemic is that I have been excluded from the church and from the sacraments. (Male, 50–55, Victoria, very frequent)

#### Faith Suffered During COVID-19

Respondents noted that church closures had affected their personal faith, but also the collective faith and practice of their community.

When it comes to religion, I feel great conflict between my belief in God and the Catholic community and what has happened and is continuing to happen in the Church … I don’t think God is listening and responding to my prayers, but feel that praying gives me access to a shared wisdom that helps me to cope with life’s challenges. (Female, 55–59, Victoria, weekly)

Since our Churches have reopened for Mass many in my age group have not returned. They are involved in other social activities but they have told me that the Mass has lost its meaning for them. I think as a Parish we have failed to keep in contact with our parishioners during CCOVID. (Female, 65–69, NSW/ACT, weekly)

Now that we are back it feels that we have lost a lot. The emotional side of things, sign of peace, no singing, no children’s liturgy, no warmth. I would rather not go at all than experience the dull non emotional Mass. I must admit I started looking at other denominations for better presentations online. (Female, 55–59, South Australia, very frequent)

#### Online Deficit

Respondents who found online worship unsatisfying generally expressed this in terms of inadequate substitution of virtual for real.

Regarding on-line communal worship, it is a poor substitute for the real thing. ... it felt all wrong—like a hollow performance instead of a collective prayer. There is also no substitute for actually taking part in communion. The sensory ritual matters—it’s not just words in your head. It felt overwhelmingly sad to be denied the Eucharist when you were most anxious. (Female, 50–54, Queensland, weekly)

I would like to add Easter was a terrible lonely time for me due to the Church closing. I did watch online but did not experience the emotions Easter Masses give me under usual circumstances. (Female, 55–59, WA, weekly)

Online Mass is no comfort to me—it’s like replacing food with cardboard, you may as well go out and eat grass. (Female, 30–34, Victoria, weekly)

Respondents distinguished between online activities that gave them spiritual enrichment, and those that did not, with most finding the online Mass less satisfying than other forms of worship.

I found more nourishment in listening to podcasts, reading reflections during Covid than watching Mass on TV/screen where I do not feel like an active participant rather just a receiver/viewer. (Female, 30–34, Western Australia, less frequent)

During the COVID-19 lockdown our parish started a weekly Gospel reflection and prayer group via Zoom that I have participated in since March 2020. ... I only watched Mass on TV/online a couple of times and ceased watching as it did not feel authentic to me. (Male, 55–59, NSW/ACT, weekly)

I did tune into virtual Masses at Easter but I don’t really like the concept much. I have found that I go online and sing hymns a lot more now. (Male, 65–69, Victoria, less frequent)

## Discussion

There is considerable diversity in these first-hand voices from Catholic pew level during COVID-19 church closures in Australia. The single largest group of responses expressed diverse views about Church and state leadership and responsibility, even though some responses praised the Catholic bishops for complying with state restrictions. Respondents very much saw Church and state working hand-in-hand, for better and for worse. This echoes McKenna’s (2021) findings among disenfranchised rural Anglicans in response to a similar UK-based survey: a sense of disappointment that the Anglican church leadership did not do more to keep churches open but was instead led by government initiatives. McKenna’s respondents also noted perceived injustices and uneven application of closures to churches as compared to other ‘non-essential’ facilities.

There were very few responses relating to the use of vaccination, masks, and social distancing, even though these were in place in most jurisdictions in Australia as churches reopened. It may be that most respondents were glad to be attending Mass in a church again and may have felt that these restrictions were a small price to pay for churches reopening. It is also worth noting the anger some respondents felt at the penetration of conspiracy theories about COVID-19 into their immediate circle of Catholic friends and family. At a time of social isolation when people were confined to very limited social circles, the sense of additional isolation must have been considerable.

The responses confirm that most Australian churchgoing Catholics adapted to a mix of both virtual and real-life forms of worship during church closures (Martyr, 2022a, 2022b). However, they provide a more detailed insight into the perceived limitations of some form of worship over others, and the persistence of belief that the Mass is best celebrated in person, rather than virtually. Respondents were frank about both the benefits and drawbacks of worshipping in new formats: while some found the overall experience enriching, others experienced the loss of community and sacraments as a form of hunger or starvation, with virtual substitutes seen as inadequate (McCarthy, 2021).

Respondents did not show a united approach either for or against church closures, and support or lack of support for this was not neatly divided along liberal/traditional lines. The most common expression of frustration was from respondents who strongly disagreed with how their fellow Catholics were behaving and believing during the pandemic, whether for or against church closures and government mandates. The sense of dismay echoes broader social disagreements during a period of immense international social polarisation over the origins and best management of COVID-19 (Bruns et al., 2020; Charron et al., 2022; Hart et al., 2020).

The internal Church divide was clearer in overall attitudes to worship, as liberal and traditional Catholics divide sharply along these lines (the other area where the division is clearest is sexual morality, Coady, 2015; Starks, 2013). Respondents who mentioned their growing interest in the Latin Mass during COVID-19 expressed views consistent with those in the current research literature on this demographic (Rymarz, 2022; Schmidinger, 2022). Similarly, more liberal Catholics have reported more positive experiences in a looser online virtual worship community (McCarthy, 2021), as was also the case here.

There are some limitations in the sample. Most Australian Catholics (98%) speak English as a first language (NCPR, 2019). However, around 36% of Australia’s churchgoing Catholics were born in countries or regions where English is not the first language (NCPR, 2020). This means that the responses above may not represent the voices of those who do not speak English fluently enough to have taken part in the original study. The original study collected data only on worship choices, religious orientation, and wellbeing. Due to time and data analysis constraints, it did not collect data on marital status, education level, and income level of participants.

## Conclusion

The Australian Catholic churchgoing population is a shrinking group and was also the group hit hardest, out of all Australian Catholics, by COVID-19 church closures. This experience was personally confronting for many of them, even without the ‘minority effect’ proposed by Clements and Bullivant (2022). Given the small size of the Australian Catholic churchgoing population, it is understandable that this is their first comprehensive qualitative COVID-19 impact study. The findings from this study support McCarthy’s (2021) themes of positive and negative experiences, including disappointment and lost opportunities. They also support McKenna’s (2021) findings in the rural Anglican community in the UK: disenfranchisement, loss of voice, and anger at church leadership’s perceived unquestioning collaboration with government initiatives.

The responses also show some polarisation around current Church politics which became woven into different COVID-19 response narratives. However, this was mostly limited to how worship took place during church closures, and how it had taken place for participants before those closures. Most of the other responses seem to have transcended the liberal/traditionalist divide.

These first-hand voices from pew level during church closures show considerable variety in response to COVID-19 church closures. While many participants found the experience enriching and were able to draw meaning from their suffering, others were angry and disheartened by loss of access to church-based worship. Future qualitative research may produce different results once Australian churchgoing Catholics have had time to reflect on and reframe their experience of church closures over 2020 and 2021.
